# Pediatric multiple sclerosis: an integrated outlook at the interplay between genetics, environment and brain-gut dysbiosis

**DOI:** 10.3934/Neuroscience.2023018

**Published:** 2023-08-23

**Authors:** Uzochukwu Adabanya, Ayoola Awosika, Anosh Khan, Ejike Oluka, Mayowa Adeniyi

**Affiliations:** 1 Anatomical Sciences, Edward Via College of Osteopathic Medicine, Monroe, USA; 2 College of Medicine, University of Illinois, Chicago, USA; 3 Emergency Medicine, Trinity health Livonia Hospital, Livonia USA; 4 Department of pathophysiology, St. George's University School of Medicine, Grenada; 5 Department of Physiology, Federal University of Health Sciences Otukpo, Benue State, Nigeria

**Keywords:** pediatric neurodegeneration, brain-gut axis, epigenetics, multiple sclerosis, neuroimmunology

## Abstract

Multiple sclerosis (MS) is a debilitating autoimmune condition caused by demyelination, neurodegeneration and persistent inflammation of the central nervous system. Pediatric multiple sclerosis (PMS) is a relatively rare form of the disease that affects a significant number of individuals with MS. Environmental exposures, such as viral infections and smoking, can interact with MS-associated human leukocyte antigens (HLA) risk alleles and influence the immune response. Upregulation of immune response results in the disruption of immune balance leading to cascade of inflammatory events. It has also been established that gut microbiome dysbiosis poses a higher risk for pro-inflammation, and it is essentially argued to be the greatest environmental risk factor for MS. Dysbiosis can cause an unusual response from the adaptive immune system and significantly contribute to the development of disease in the host by activating pro-inflammatory pathways that cause immune-mediated disorders such as PMS, rendering the body more vulnerable to foreign attacks due to a weakened immune response. All these dynamic interactions between biological, environmental and genetic factors based on epigenetic study has further revealed that upregulation or downregulation of some genes/enzyme in the central nervous system white matter of MS patients produces a less stable form of myelin basic protein and ultimately leads to the loss of immune tolerance. The diagnostic criteria and treatment options for PMS are constantly evolving, making it crucial to have a better understanding of the disease burden on a global and regional scale. The findings from this review will aid in deepening the understanding of the interplay between genetic and environmental risk factors, as well as the role of the gut microbiome in the development of pediatric multiple sclerosis. As a result, healthcare professionals will be kept abreast of the early diagnostic criteria, accurately delineating other conditions that can mimic pediatric MS and to provide comprehensive care to individuals with PMS based on the knowledge gained from this research.

## 1. Introduction

Multiple sclerosis (MS) is a chronic inflammatory autoimmune disease that leads to neuro-axonal degeneration, causing extensive damage to the brain and spinal cord nerves [1]. The disease progresses over time, with symptoms becoming more intense and affecting speech and movement abilities [1]. It usually manifests in a relapsing and remitting pattern, with periods of clinical presentation and recovery [2]. Some patients experience minor symptoms, while others may suffer from impairment due to partial recovery from relapses or natural disease progression [1]. MS is a leading cause of disability in young adults, but it is not commonly seen in individuals under the age of 16 [2]. However, several studies have found that at least 5% of individuals with MS are children [3], with the highest prevalence being among those aged 13 to 16. Although the exact cause of MS is unknown, the aim of this review is to explore the cross integration between brain-gut-dysbiosis, genetics and environmental factors as contributory factors to the development and complications of pediatric MS. This will also keep healthcare practitioners aware of early diagnostic criteria, accurately delineating other conditions that can mimic pediatric MS and management strategies. Despite extensive research, there is currently no cure for MS. Treatment options focus on managing and alleviating symptoms and can include relapse treatment and immune-modulatory therapy for pediatric patients [4]. The role of exercise rehabilitation/prescription strategies are rarely discussed in Pediatric MS patients. This promising non-pharmacologic approach will also be elucidated.

Due to the unpredictable nature of the disease and the wide range of available treatments, clinicians may struggle to educate patients and their families, which makes them more anxious and uncertain. Hence, children with MS often require an interdisciplinary approach to treatment, involving collaboration from a range of healthcare professionals, such as pharmacologists, physiotherapists, nurses, case workers, community health workers, psychologists and physicians, including neurologists, ophthalmologists and, in some cases, psychiatrists [5].

## 2. Methodology

A narrative literature search was carried out using web-based databases like Web of Science, Pubmed (the National Library of Medicine of the USA, which contains a database of medical and biomedical research literature), Google Scholar, EMBASE (the Excerpta Medica Database) and Scopus. MeSH terms used to guide search strategy were formed using the PICO (patient/population, intervention, comparison and outcome) criteria for evidence-based medicine investigations. The search term adopted includes “pediatric or child or adolescent and multiple sclerosis”, “brain-gut axis, environmental or genetic”, “treatment modalities or rehabilitation” and “quality of life”. Relevant articles were filtered based on set inclusion and exclusion criteria, followed by a manuscript review by two authors and a third party for any disagreements.

## 3. Review

### 3.1. History and Epidemiology of MS

Research has revealed that MS has a significant impact on millions of individuals globally and is recognized as the primary cause of non-traumatic impairment in young adults [6]. The existence of a progressive condition akin to MS has been documented since the late 1300s [7], but it was not until 1868 that neurologist Jean-Martin Charcot described its symptoms [8] and coined the term “la sclérose en plaques” [9]. As a neuroscientist at the Hôpital de Salpétrière, Charcot differentiated MS from the tremors of paralytic agitans, which later became known as Parkinson's disease. Charcot identified three key symptoms of MS, including intention tremor, nystagmus and scanning speech [10], which were later called “Charcot's multiple sclerosis triad”.

The symptoms of this immune-mediated demyelinating illness vary greatly and are difficult to predict [11]. Detecting, characterizing and treating the disease can be challenging due to these diverse presentations. MS generally affects women more than men, with almost double the number of women affected than men, except for those with the primary-progressive type [2],[3],[11]. Initial symptoms are rare before the age of ten or after the age of sixty [12]. Since 1985, there has been a noticeable increase in the number of MS cases, particularly among women [13], but this is partly due to improved detection methods. Studies have shown that estimates of pediatric MS cases vary depending on geographic location, with a link established between MS diagnosis and distance from the equator [14]. It has also been found that at least 5% of individuals with MS are children [3], with the highest prevalence being among those aged 13 to 16 [3],[14]. Another distinguishing feature in geographical distribution is exemplified by the statistics that half of all MS patients are from Europe, and the disease is more common in areas with higher socioeconomic status [5]. Therefore, geographic and socioeconomic factors significantly influence the incidence and prevalence of pediatric MS [14].

### 3.2. Risk factors for MS

#### 3.2.1. Normal gut microbiome

The gastrointestinal (GI) microbiome is an exceptional and dynamic system that carries out a range of beneficial functions. These functions are crucial and involve defending the host against pathogenic colonization, fortifying the intestinal barrier, breaking down nutrients and dietary fiber and enhancing the host's immune system [15]. Furthermore, the commensal microbes in this microbiota provide significant vitamins to the host, including biotin, cobalamin, riboflavin, vitamin K and folates [15],[16]. The microbiota of the GI system is originally acquired from the mother during childbirth and is subsequently altered by factors such as genetic makeup, diet, infection, antibiotic usage, stress, hygiene and aging [17]. The microorganism population in the GI tract is substantially larger (up to 100 trillion) than other microbial communities found on the body's surfaces and is roughly 10 times greater than the sum of our somatic and germ cells [17],[18]. The functional attributes that we possess without evolving ourselves are bestowed upon us by the gut microbiome, which may contain more than 300 times the number of genes present in our own genome [18]. The gut microbiota comprises around 1,000 to 1,500 bacterial species. Nevertheless, an individual typically possesses only about 160 bacterial species [17]. This demonstrates that the microbiome's configuration varies greatly between people and is influenced by both environmental factors and genetic inheritance [19],[20]. Typically, Bacteroidetes and Firmicutes are the primary phyla found in the gut of a healthy individual, while Cyanobacteria, Proteobacteria, Actinobacteria, Verrucomicrobia and Fusobacteria are present in lesser amounts [21]. The microbial communities in the different sections of the GI tract vary, with gram-positive bacteria being more common in the small intestine and gram-negative bacteria being more prevalent in the large intestine [22].

#### 3.2.2. Gut-brain axis: Microbiome dysbiosis and its role in Pediatric MS

The development of gut-associated lymphoid tissues (GALT), in addition to the normal development of humoral and cellular components of the mucosal immune system, is significantly influenced by the gut microbiota [23],[24]. The innate immune system's hematopoietic and non-hematopoietic cells recognize signaling molecules and metabolites released by commensal microbes, leading to various physiological responses [25]. The development of mucosal immunity is significantly influenced by the intestinal microbiome, as depicted by studies done on germ-free (GF) mice. In contrast to animals that are specifically pathogen-free (SPF), animals raised in a GF environment have been found to generate a lower number of intraepithelial lymphocytes (IELs) [26], have a notable decrease in IgA-secreting plasma cells within the lamina propria, have a reduced presence of regulatory T (Treg) cells and have a significantly lower mRNA expression level of Ang4 [27]. Based on the findings, mucosal immunity necessitates the presence of gut microbiota. Specific commensal bacteria, such as Clostridia species, along with the short-chain fatty acids (SCFAs) they generate, encourage the expansion of colonic Treg cells. These cells restrict inflammation and maintain intestinal balance [28],[29]. Nonetheless, even a minor shift in the makeup of the gut microbiota can disrupt immune balance, resulting in various inflammatory conditions and infections [30].

Dysbiosis, which refers to an imbalanced gut microbiome, results in changes in the ratio of beneficial bacteria to pathogenic ones, and the translocation of colonic bacteria [31]. Dysbiosis can cause an unusual response from the adaptive immune system and significantly contribute to the development of disease in the host by activating pro-inflammatory pathways that cause immune-mediated disorders such as type 1 diabetes, rheumatoid arthritis and MS [30]. Dysbiosis has also been shown to promote the growth of cancerous cells by rendering the body more vulnerable to foreign attacks due to a weakened immune response [30],[32]. A variety of factors such as dietary changes, vitamin D deficiency, infections, vaccination, antibiotic exposure and birth mode contribute to dysbiosis. The impact of microbiota on immune cell development and the nervous system cannot be over emphasized as it stands to be a major contributor of host-microbe interaction, dictating the course of central nervous system autoimmune dysfunction seen in PMS [32].

The integrative method of the gut-brain axis involves afferent and efferent neural, endocrine, metabolic and immunological pathways [33]. The human gut microbiota synthesizes important vitamins that contribute to the synthesis of neurotransmitters like serotonin, histamine, dopamine and Gama-aminobutyric acid. This strong communication between the gastrointestinal, immune and nervous systems also comes as no surprise, as dysbiosis of gut microbiota plays a major role in various central nervous systems disorders, including PMS [34]. Some scientists have also postulated an important role microbiota may have in modulating pain, behavioral patterns and overall modulation of brain chemistry [35],[36].

The dynamic interaction between these systems culminates into a chronic inflammatory state that leads to demyelination and degeneration of neuro-axons. Although there are several risk factors involved in the development of autoimmune disorders, the greatest environmental risk factor for MS is argued to be the gut microbiome [36]. Experimental autoimmune encephalomyelitis (EAE) is the most popular animal model utilized to study human MS and is, in fact, the most thoroughly researched model of autoimmunity. EAE may be initiated by introducing bacterial adjuvants in conjunction with certain antigens, including myelin basic protein (MBP), proteolipid protein (PLP) or myelin oligodendrocyte glycoprotein (MOG). This activates T cells that particularly attack myelin, leading to the distinct pathological alterations that are linked to the disease process [37],[38].

Tremlett and colleagues aimed to investigate the differences in gut microbiota between children with and without MS and examine the connection between MS relapse and gut microbiota. Although the first examination did not reveal any significant differences in the makeup of the microbiota, a more thorough analysis at the lower taxonomic ranks showed considerable variations, with MS-afflicted children having an Actinobacteria abundance that was 2.5 times greater than that of non-MS children [39]. Actinobacteria has been shown to be associated with additional conditions that involve inflammation, such as inflammatory bowel disease, which is a noteworthy observation [40]. These study findings also showed a heightened risk of on-study relapse linked to the nonexistence of Fusobacteria, an increased prevalence of Firmicutes and the existence of Euryarchaeota. The study demonstrated that children with MS may have a gut microbiome that promotes inflammation. Additionally, it was also suggested that the gut microbiota may affect the immune system, which may lead to MS relapses [40].

#### 3.2.3. Environmental factors and its role in Pediatric MS

**Figure 1.**
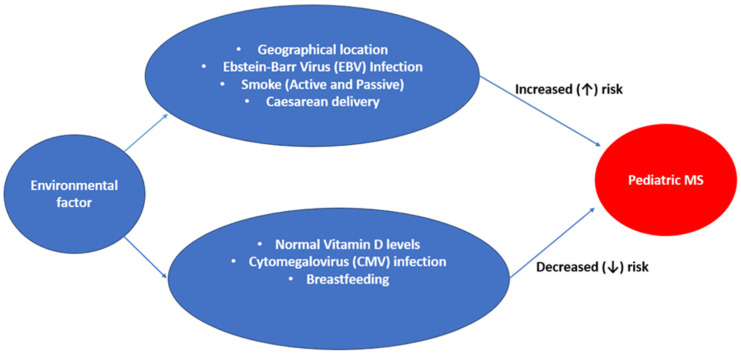
Showing various environmental factors that play a role in MS.

An array of environmental factors (Figure 1) has shown some link to the progression of pediatric MS. Some of the pathophysiologic mechanisms underlying these factors involve modulation of immune response and genetic dysregulation. Hence, analyzing pediatric MS cases offers a unique opportunity to investigate early-life exposures, particularly as these patients are closer in time to the biological events believed to contribute to the disease's onset [41].

On a global scale, there is a correlation between the prevalence of MS and latitude that cannot be explained by the distribution of high-risk genetic alleles [42]. Individuals who relocate from high-risk regions to lower-risk areas before the age of 16 have a reduced risk of developing the disease. In contrast, those who relocate from a low-risk region to a high-risk region retain the lower risk of their original location. Nevertheless, their descendants exhibit a risk level comparable to that of the new country they inhabit [43]. There is an inverse correlation between the prevalence of MS and the average number of hours of sunshine exposure per year [36], as ultraviolet radiation exposure helps with the synthesis of Vitamin D. Additionally, individuals with MS who reside in regions with high sunlight exposure and engage in outdoor work have been linked with decreased mortality rates [44].

In both pediatric and adult patients with MS, the seroprevalence rates of distant EBV infection are higher when compared to healthy control subjects, as per studies [45]. On the other hand, remote CMV infection was shown to be linked to fewer PMS diagnoses as indicated by the existence of positive serologic anti-CMV IgG [46]. Smoking increases the risk of MS. This risk is further multiplied in a dose-dependent manner in persons with a higher cumulative smoking dose. According to a case-control study conducted in France, there is a significant correlation between the frequency of exposure to passive smoking from parents and an elevated risk of developing MS [47]. The way infants are born has generated contradictory findings in studies, as some indicate that having a Caesarean delivery could raise the likelihood of MS, while others report no connection between delivery method and risk [48]. Breastfeeding in infancy could potentially lower the likelihood of developing pediatric-onset MS later in life [49].

#### 3.2.4. Genetics factors and its role in Pediatric MS

It is currently uncertain whether the genetic factors identified in adults with MS can be applied to children with MS. Nevertheless, there is evidence indicating that the same genetic risk factors may be shared between pediatric and adult-onset MS as represented in Table 1 [41]. To explore the association between HLA-DRB115 status and the risk of developing MS in children with acquired demyelinating syndrome, a study was conducted with 266 participants. The results showed that children with HLA-DRB115 alleles were more likely to be diagnosed with MS in the future, particularly those of European ancestry. However, there was no difference in DRB115 allele frequency between children with monophasic acute demyelinating syndrome and unaffected control subjects [50], indicating that the HLA-DRB115 allele is a risk factor for pediatric MS but not for monophasic demyelinating events [51].

A weighted genetic risk score was used to evaluate the possibility of similar risks posed by non-HLA SNPs in pediatric MS patients, which were discovered through GWAS [41]. In a more extensive pediatric cohort (comprising over 700 patients who developed MS before the age of 18), a recent GWAS study discovered that various non-MHC SNPs were significantly associated with pediatric-onset MS. Furthermore, several of these SNPs had a more significant effect in comparison to GWAS results in adults [52].

The examination of genetic variations in the mitochondria has been investigated in children who have acquired demyelinating syndromes (ADS) as well [41]. One preliminary investigation found specific mitochondrial haplogroups were related to ADS. Furthermore, a particular haplotype was connected to a higher risk of multiple sclerosis (MS) after a first acquired demyelinating event [53].

**Table 1.** shows alleles and their temporal relationship with MS.

**Allele**	**Temporal relationship**
HLA DRB1*15:01	Increased risk
HLA A*02	Decreased risk
Non-HLA SNPs	Increased risk

*HLA- human leukocyte antigen, SNPs- single nucleotide polymorphisms*.

#### 3.2.5. Role of Epigenetics in Pediatric MS: Multifaceted interaction

Scientists in the field of epigenetics have found that the white matter of the central nervous system in a MS subject experience an upregulation in peptidylargininedeiminase-2 (PAD2) enzyme, which produces a less stable form of myelin basic protein and ultimately leads to the loss of immune tolerance [54]. These mechanisms can be influenced by a range of factors, including biological, environmental and hereditary factors [41]. Additional research has indicated that genes like IL-13, IL-17, *IFNG* and *FOXP3*, which are responsible for mediating immune system and T-cell differentiation, may be subject to hypomethylation, resulting in their increased expression [55]. Conversely, hypermethylation of genes involved in immune system regulation, such as SHP-1, can lead to decreased genetic expression and an increase in leukocyte-mediated inflammation [56]. Environmental exposures like viral infections and smoking can interact with MS-associated HLA risk alleles, influencing the immune response. For instance, early-life herpes simplex virus-1 infection appears to be protective in HLA-DRB115 positive individuals [46], while smoking significantly increases the risk of MS in HLA-DRB115 positive individuals who are negative for the HLA-A02 allele [57]. HLA risk alleles have also been linked to obesity, with obese persons positive for HLA-DRB115 and negative for HLA-A*02 exhibiting a higher chance for developing MS [57].

Finally, gut microbiota is also capable of producing metabolites that can promote epigenetic modifications capable of impacting immune regulation and neuroinflammation in pediatric MS [36],[57]. Dysbiosis, or perturbation in the commensal microbial population, is frequently associated with an increased intestinal permeability, or “leaky gut”, which allows the entrance of exogeneous molecules, in particular bacterial products, and metabolites, that can disrupt tissue homeostasis and induce inflammation, promoting both local and systemic immune responses [37]. An altered gut microbiota could therefore have significant repercussions not only on immune responses in the gut but also in distal effector immune sites such as the CNS [39],[56].

## 4. Clinical symptoms and manifestations of Pediatric MS

MS in children typically begins with an acute demyelination episode. Pediatric MS can present as a clinically segregated syndrome or, in rare cases, as acute disseminated encephalomyelitis, like in adult MS. Notably, the clinically isolated syndrome is inferred when medical manifestations of the first episode last more than one day without any indication of encephalopathy but with probable inflammatory neurodegeneration [58].

Most children with pediatric MS manifest as relapsing-remitting MS, with the number reaching as high as 85 to 100% in several studies, which is lower in adult MS [59]. The higher relapse rate observed in PMS means there is usually a short interval between the first attack and a subsequent demyelinating event [60]. Most patients recover completely, but a few develop residual disabilities from incomplete recovery after a relapse [61]. It is widely observed that children have a slower rate of progression to irreversible disability, which takes about 10 years longer to reach when compared to adult MS, but overall, they achieve this milestone at a much younger age [60]. MS disease progression ranges from mild with a relatively modest handicap after a lengthy duration of the illness to a malignant variant with severe impairment or death within only a few months of disease onset [58].

Pediatric MS may present with varying features such as optic neuritis, transverse myelitis, sensory loss and bladder dysfunction [59]. Prepubertal children often demonstrate polyfocal symptoms that involve sensory, cerebellar, visual, brainstem or pyramidal changes [62]. Encephalopathy and seizures are also typically common in PMS compared to their adult counterparts [62].

Cognitive impairment has been reported in up to a third of patients with PMS, and the domains affected include information processing speed, memory, attention, executive function, visual perception and language functioning [59]. This has been linked to a disruption of CNS development that impairs the maturation of neuronal pathways involved in cognitive functioning [59].

### 4.1. Complications resulting from Pediatric Multiple Sclerosis

In aggressive degenerative neurological lesions, children have a more significant clinical presentation than adults, and this results in multi-system complications depending on the region of the nervous system affected [63]. The condition can lead to mobility impairment, fatigue, vision problems and coordination difficulties. Emotional and behavioral consequences, such as depression, sexual dysfunction and social isolation, are also common [63]. Medication-related side effects and challenges in medication adherence further contribute to the complexity. Paresthesia and partial loss of sensation are prevalent and frequently localized to one or both hands and legs. Burning and electric shock-like sensations might occur spontaneously or in reaction to contact with spinal cord lesions. Early in the illness, objective sensory abnormalities may be temporary and difficult to detect [63]. All these can negatively influence the career and social abilities of patients with pediatric MS.

## 5. Diagnosis and diagnostic criteria of Pediatric MS

Eliciting diagnosis of PMS can be difficult because of the presence of many conditions that can mimic PMS in this age group. A careful evaluation is required to exclude other demyelinating conditions that may present similarly. Various diagnostic tools, including magnetic resonance imaging (MRI), are used to identify changes in the central nervous system (CNS). MRI is an important diagnostic tool for MS as it can detect inflammatory plaques in the CNS and can be performed easily in children. It is also useful in monitoring disease progression. To diagnose MS, specific MRI findings must be present, such as hyperintense (T2-weighted) lesions in at least one of the four CNS regions, indicating dissemination in space, and the presence of silent gadolinium-enhancing and non-enhancing lesions, indicating dissemination in time [62].

CSF analysis is another useful tool for evaluating children with demyelinating symptoms. Oligoclonal bands and the IgG index are typically elevated in MS [63] and can differentiate it from acute demyelinating encephalomyelitis (ADEM), a common MS mimic. Additional investigations include serum studies to identify antibodies specific to MS mimics, such as anti-aquaporin-4 antibodies for neuromyelitis optica spectrum disorder (NMOSD) and an antinuclear antibody (ANA) panel for rheumatologic conditions [62],[63]. The International Pediatric Multiple Sclerosis Study Group (IPMSSG) criteria, developed in 2007 and revised in 2013 as listed in Table 2, are widely used to diagnose pediatric MS and other acute demyelinating syndromes that can resemble MS in this age group [64]. An update to the criteria is expected that will incorporate the 2017 update to McDonald criteria for multiple sclerosis to it [65].

**Table 2.** Showing the diagnostic criteria for pediatric CIS, ADEM, NMO and MS [64].

**Pediatric Multiple Sclerosis**	**Diagnostic Criteria**
***Pediatric Neuromyelitis Optica (NMO)***	***All must be present*** Acute myelitis Optic neuritis At least two of the three supportive criteria are: o Contiguous spinal cord MRI lesion extending over 3 vertebral segments o Brain MRI does not meet the diagnostic criteria for multiple sclerosis. o Aquaporin-4 IgG seropositive status.
***Pediatric Clinically Isolated Syndrome (CIS)***	***All must be present*** A monofocal or polyfocal clinical CNS event with a presumed inflammatory demyelinating cause. Absence of a prior clinical history of CNS demyelinating disease (e.g., absence of previous optic neuritis, transverse myelitis, or hemispheric or brainstem-related syndromes). No encephalopathy (i.e., no changes in consciousness or behavior) that cannot be explained by fever. The diagnosis of MS based on baseline MRI features is not met.
***Pediatric Acute Disseminated Encephalomyelitis (ADEM)***	***All must be present*** A first-ever polyfocal, clinical CNS event with a presumed inflammatory demyelinating cause. Encephalopathy that cannot be explained by fever. No new clinical or MRI findings emerge 3 months or more after onset. Abnormal brain MRI during the acute (3-month) phase, which typically reveals: o Diffuse, poorly demarcated, large (>1–2 cm) lesions involving predominantly the cerebral white matter. o Deep gray matter lesions (e.g., thalamus or basal ganglia) can be present. o T1 hypointense lesions in the white matter are rare.
***Pediatric Multiple Sclerosis (can be satisfied by any of the following)***	2 or more CIS separated by more than 30 days and involving more than one area of the CNS. One CIS associated with MRI findings consistent with the 2017 Revised McDonald criteria for dissemination in space (DIS) and in which a follow-up MRI shows at least 1 new lesion consistent with dissemination in time (DIT) MS criteria. One ADEM attack followed by one CIS, 3 or more months after symptom onset, that is associated with new MRI lesions that fulfill the 2010 Revised McDonald DIS criteria4. A first, single, acute event that does not meet ADEM criteria and whose MRI findings are consistent with the 2017 Revised McDonald criteria for DIS and DIT (applies only to 11-year-old children).

ADEM: Acute Demyelinating Encephalomyelitis, CIS: Clinically Isolated Syndrome, CNS: Central Nervous System, DIS: Dissemination In Space, DIT: Dissemination In Time, MRI: Magnetic Resonance Imaging, NMO: Neuromyelitis optica, ON: Optic Neuritis, TM: Transverse Myelitis

### 5.1. Differential diagnosis of Pediatric MS

Table 3 provides an extensive list of potential causes for pediatric MS, ranging from autoimmune disorders to infectious processes and metabolic diseases. However, there are certain factors that may suggest a diagnosis other than MS in a child who presents with an acute demyelinating episode. These include the presence of fever, encephalopathy, a very young age at presentation, a progressive course of events and other systemic involvements, such as the peripheral nervous system. If the child's cerebrospinal fluid analysis shows no oligoclonal band or a significant increase in white blood cells, alternative diagnoses should be considered [2],[63]. It is important to note that the list of potential causes for pediatric MS is extensive and not exhaustive [2].

ADEM is the common disease entity that mimics MS and differentiating it from MS can be difficult. It usually begins after a viral illness or vaccination [63], is common in young children and presents as encephalopathy and multifocal neurologic symptoms with a monophasic course [66]. MRI reveals lesions involving the white mater and gray mater that appear as diffuse, asymmetric and poorly demarcated hyperintensities [20],[66]. Although most symptoms resolve spontaneously within a few months, a study found that 6% to 18% of affected children will have future relapses that meet the criteria for MS [63].

NMOSD is another demyelinating condition that primarily affects the optic nerves and the spinal cord [67]. Disease onset is usually around the age of 10 and a slightly female predominance has been noted. This condition is mediated by antibodies against the aquaporin-4 water channel (AQP4), which is a protein found on astrocytes. These autoantibodies can be detected in the serum and CSF that aids in the diagnosis. Distinct MRI features have been noted on imaging studies. Careful consideration to exclude this condition before commencing MS treatment is paramount because studies have shown worsening of the NMOSD symptoms with some of the therapies used in MS like B-interferon, fingolimod, natalizumab, etc. [63].

Myelin oligodendrocyte glycoprotein associated disease (MOGAD) is an inflammatory demyelinating disease of the CNS that occurs in the presence of MOG antibodies [68]. MOG is a glycoprotein found on the outer surface of the CNS myelin sheath and on the membrane of the oligodendrocytes. It can present in a monophasic or relapsing pattern. MOG antibodies have been isolated in other pediatric demyelinating conditions like optic neuritis, transverse myelitis and ADEM [63],[69]. The presence of MOG antibodies argues against a diagnosis of MS [69]. It is very important for clinicians to appropriately exclude these potential confounding diseases that can mimic pediatric MS.

**Table 3.** Showing differential diagnosis of pediatric MS [63],[64].

**Diagnosis of Pediatric MS**	**Details**
Leukodystrophy	Metachromatic leukodystrophy, Krabbe disease, Adrenoleukodystrophy, Refsum disease, Wilson's disease, Fabry disease.
Infectious	Bacterial: Syphilis, Lyme disease (Neuroborreliosis), mycoplasma, post-streptococcal, Listeria, Bartonella, Whipple disease. Viral: HIV, HSV, EBV, HTLV, JCV/PML. Fungal: Cryptococcus, Histoplasmosis, Blastomycosis, Coccidomycosis. Parasitic: Neurocysticercosis.
Demyelinating	ADEM, Optic neuritis, Transverse myelitis, NMOSD, MOG-AD, CRION, Guillian-Barre syndrome.
Inflammatory	SLE, Sjogren syndrome, Neurosarcoidosis, Antiphospholipid antibody syndrome, Behcet disease.
Mitochondrial	MELAS, MERRF, LHON, NARP, Leigh syndrome, Kearn-Sayre syndrome
Genetic/metabolic	Amino Acidurias, Inborn errors of metabolism, Hemophagocytic lymphohistiocytosis.
Neoplastic	Glioma, Astrocytoma, CNS lymphoma, Ependymoma, Medulloblastoma, Metastases.
Vascular	CADASIL, Moya Moya disease, Carotid dissection, DVST, Primary angitis of CNS, arterial ischemic stroke.
Endocrine	Thyroid disease, Diabetes mellitus.
Nutritional	Vitamin B12, B9 (folate) and vitamin E deficiencies.
Toxin/drug induced	Central pontine myelinosis, Amphetamine, Radiation, Chemotherapy (Cyclosporine, Cytosine-arabinoside).

ADEM: acute disseminated encephalomyelitis, CNS: central nervous system, CRION: chronic inflammatory relapsing optic neuropathy, DVST: deep vein sinus thrombosis, EBV: Epstein barr virus, HIV: human immunodeficiency virus, HSV: herpes simplex virus, JCV: JC virus, LHON: lebers hereditary optic neuropathy, MELAS: mitochondrial encephalopathy with lactic acidosis and stroke-like episodes, MERRF: myoclonic epilepsy with ragged red fibers, MOGAD: myelin oligodendrocyte glycoprotein associated disease, NMOSD: neuromyelitis optica spectrum disorder, NARP: neuropathy ataxia and retinitis pigmentosa, PML: progressive multifocal leukoencephalopathy

## 6. Treatment and management of Pediatric MS

Treatment of pediatric MS patients relies principally on case series, general agreement and established guidelines. The lack of established information limits our knowledge of treatment effectiveness. Studies on interferon and glatiramer acetate have shown a decrease in relapses post-treatment, and more consistently, a commonly promising safety profile. Notwithstanding, many patients with pediatric MS on these treatments still present with new relapses and seem to accumulate new lesions that indicate the need for a change from first line to second-line treatments [2],[70].

A recent randomized double-blinded clinical trial demonstrated an 82% reduction in annual relapse rate in patients taking fingolimod compared to those taking interferon beta. Pediatric patients taking fingolimod also had a reduction in MRI metrics. There was a reduction in the rate of brain atrophy or appearance of new lesions in those taking fingolimod compared to those on interferon (−0.48 versus −0.80 respectively) [65],[70]. Patients on fingolimod, however, presented with more serious side effects such as leukopenia, seizures and hypersensitivity reactions.

Dimethyl fumarate is another drug commonly used. After a 24-week intervention, there was a significant reduction in the incidence of T2 hyperintense lesions seen [2],[65]. Patients on dimethyl fumarate also presented with side effects that were mostly gastrointestinal with some flushing; however, severe adverse effects were not seen. There have also been clinical trials of teriflunomide and alemtuzumab (CD52 antibody). These trials are still ongoing and set to be completed in 2015 [65].

B cell target therapies, such as Rituximab, have been used in many other pediatric autoimmune conditions. This therapy has also been recognized due to their ability to influence B cell interactions with T cells and the reduced side effect profile especially when used in isolation [2],[65]. Ocrelizumab, the only FDA-approved medication for primary progressive MS in adults is also beginning to be recognized as a form of treatment in patients with a relapsing course [65],[71].

Natalizumab (Anti alpha 4 integrin antibody) can be employed in refractory cases. In a large study in Italy, 101 patients with pediatric MS were treated with monthly infusions, and a total of 15 relapses was seen with 9 patients over an average treatment interval of 34 months [65]. A retrospective study done in Germany had 40% of their 144 patients who were categorized as having highly active MS based on study criteria. These patients established improved relapse frequencies with a reduction in MRI markers of disease activity while taking both fingolimod and natalizumab [65],[72]. The study also noted a trend toward greater response to natalizumab [65],[72].

While an all-inclusive debate concerning safety for these evolving therapies is outside the scope of this analysis, it is of important that clinicians recommending the drugs presented in Table 4, be fully conversant of the pertinent risks, pre-treatment valuations, therapy approvals, observation of risks, conception implication and drug interactions.

### 6.1. Disease modifying therapies

**Table 4.** Showing a list of disease modifying therapy.

**Drug**	**Dosing**	**Adverse Effects**	**Annual relapse rate (24)**
Interferon beta-1a [2]	30 mcg once per week, IM. 22 or 44 mcg 3 times per week, Subcutaneous (SC).	Flu-like illness, headache, elevated liver enzymes, injection site reaction.	0.47
Interferon beta-1b [2]	250 mcg every other day, SC.		
Glatiramer acetate [1]	20 mg daily, SC OR 40 mg, 3 times per week, SC	Post-injection reactions: palpitations, flushing, laryngeal constriction, dyspnea, anxiety; injection site pain, redness, or itching.	0.31
Fingolimod [1]	0.5 mg once per day, oral.	Bradycardia with first dose, increased susceptibility to infections (HSV, VZV, Cryptococcus), PML, macular edema, abnormal liver transaminases.	0.16
Daclizumab [4]	150 mg once per month, SC.	GI symptoms, hepatotoxicity, increased infection risk, skin reactions.	0.45
Dimethyl fumarate [4]	240 mg twice per day, oral.	Angioedema, anaphylaxis, GI symptoms, flushing, lymphopenia, abnormal LFTs, PML.	0.44 to 0.49
Teriflunomide [4]	7 mg or 14 mg once daily, oral.	Hair thinning, teratogenicity, hepatotoxicity, GI upset, CYP1A2 inducer.	0.32 to 0.39
Natalizumab [2]	300 mg once per month, IV.	PML, liver injury, hypersensitivity reaction, headache.	0.22
Rituximab [1]	375 mg/m^2 weekly for 4 weeks, IV.	Allergic reaction, increased risk of PML, severe hepatitis B reactivation.	
Ocrelizumab [5]	First 2 doses of 300 mg, separated by 2 weeks, followed by 600 mg every 6 months, IV.	Infusion-related reactions, increased risk of infection, malignancy.	0.50
Cyclophosphamide [5]	600-1000 mg/m^2 per dose, IV or oral. Dose titration per patient lymphocyte count.	Bone marrow toxicity, hepatotoxicity, hemorrhagic cystitis, bladder cancer, alopecia, amenorrhea, infertility, GI symptoms.	-
Mitoxantrone [9]	12 mg/m^2 once every 3 months, IV.	Dose-dependent cardiomyopathy, acute promyelocytic leukemia.	0.30 (25)
Alemtuzumab [9]	12 mg daily for 5 days, followed in a year by 12 mg daily for 3 days, IV.	Secondary autoimmune thyroid disorders, autoimmune cytopenias (e.g., ITP), increased susceptibility to infections, infusion reactions.	0.18 to 0.26

IM- intramuscular, SC- subcutaneous, IV- intravenous; mcg- microgram, mg- milligram, HSV: herpes simplex virus, VZV- varicella zoster virus, PML: progressive multifocal leukoencephalopathy, LFT- liver function test, ITP: immune thrombocytopenic purpura

### 6.2. Role Exercise prescription as an adjunct treatment

Exercise has been used as part of a multidisciplinary approach rarely discussed for managing Pediatric Multiple Sclerosis (MS) patients. Recent research suggests that exercise can actually improve symptoms of fatigue and disability, despite earlier concerns that it may worsen the patient's condition. Different types of exercise have been tested in adults with MS, including resistance training, aquatic therapy and aerobic exercise. Aquatic exercises have been shown to be particularly effective due to the cooling effect of water that can reduce the worsening of MS symptoms associated with high body temperature, also known as the Uhthoff phenomenon [73]. It is important for those with MS to perform low-impact and low-intensity cardiopulmonary exercises with enough recovery time between sessions. According to American College of Sports medicine guidelines, the recommended exercise protocol for individuals with MS consists of engaging in aerobic activity for 10 to 30 minutes at moderate intensity for two to three days per week and participating in resistance training by performing one to three sets of 8-15 repetition maximum (RM) for two to three days per week [73]. Although further investigation is required to establish the efficacy of exercise therapy in treating pediatric MS, it has the potential to considerably enhance the quality of life of MS patients in the future.

## 7. Conclusion

MS is an autoimmune disease that affects the nervous system and is the primary cause of physical and cognitive disability in young people, significantly impacting their quality of life. However, pediatric MS has historically been overlooked and undertreated, despite having distinct characteristics and a different disease history than adult-onset MS. Children's neuroplasticity may slow down disease progression, but impairment can still occur at an earlier age. Treatment for MS should prioritize medications that can modify the course of the disease, such as immune-modulatory therapies, which are classified as first-line or second-line immunotherapeutic treatments. It is recommended that pediatric MS patients start taking these disease-modifying medications as soon as possible, as they are essential for managing relapsing-remitting MS. Different treatment options aim to reduce short-term MRI lesion activities, with long-term objectives, including secondary progressive MS prevention. Patient compliance and monitoring for medication toxicity are key concerns following the start of therapy. Pediatric MS can significantly impact various aspects of a child's life, including education, emotions, socialization and physical well-being, making early detection and proper management essential. Pediatric MS provides a unique chance to investigate potential triggers or modifiers of the disease. The close monitoring provided by parents and caregivers can also facilitate the collection of accurate lifestyle and related exposure information. Additionally, since individuals with pediatric MS have lived fewer years, there is a greater opportunity to obtain clinical and biological information in close proximity to the actual onset of the disease compared to adults. Therefore, it is important to conduct further research to thoroughly examine these factors, which will provide reliable information to patients and their families.
